# Optimizing MRI sequence classification performance: insights from domain shift analysis

**DOI:** 10.1007/s00330-025-11671-5

**Published:** 2025-05-26

**Authors:** Mustafa Ahmed Mahmutoglu, Aditya Rastogi, Gianluca Brugnara, Philipp Vollmuth, Martha Foltyn-Dumitru, Felix Sahm, Stefan Pfister, Dominik Sturm, Martin Bendszus, Marianne Schell

**Affiliations:** 1 Department of Neuroradiology, Heidelberg University Hospital, Heidelberg, Germany; 2 Department of Neuroradiology, Bonn University Hospital, Bonn, Germany; 3 Division for Computational Radiology and Clinical AI, University Hospital Bonn, Bonn, Germany; 4 Division for Medical Image Computing, German Cancer Research Center (DKFZ), Heidelberg, Germany; 5 Hopp Children’s Cancer Center (KiTZ), Heidelberg, Germany; 6 Department of Neuropathology, Heidelberg University Hospital, Heidelberg, Germany; 7 Clinical Cooperation Unit Neuropathology, German Cancer Research Center (DKFZ) and German Cancer Consortium (DKTK), Heidelberg, Germany; 8 Division of Pediatric Neurooncology, German Cancer Research Center (DKFZ) and German Cancer Consortium (DKTK), Heidelberg, Germany; 9 Division of Pediatric Glioma Research, German Cancer Research Center (DKFZ), Heidelberg, Germany; 10 Department of Pediatric Oncology, Hematology & Immunology, Heidelberg University Hospital, Heidelberg, Germany

**Keywords:** Deep learning, Convolutional neural networks, Magnetic resonance imaging

## Abstract

**Background:**

MRI sequence classification becomes challenging in multicenter studies due to variability in imaging protocols, leading to unreliable metadata and requiring labor-intensive manual annotation. While numerous automated MRI sequence identification models are available, they frequently encounter the issue of domain shift, which detrimentally impacts their accuracy. This study addresses domain shift, particularly from adult to pediatric MRI data, by evaluating the effectiveness of pre-trained models under these conditions.

**Methods:**

This retrospective and multicentric study explored the efficiency of a pre-trained convolutional (ResNet) and CNN-Transformer hybrid model (MedViT) to handle domain shift. The study involved training ResNet-18 and MedVit models on an adult MRI dataset and testing them on a pediatric dataset, with expert domain knowledge adjustments applied to account for differences in sequence types.

**Results:**

The MedViT model demonstrated superior performance compared to ResNet-18 and benchmark models, achieving an accuracy of 0.893 (95% CI 0.880–0.904). Expert domain knowledge adjustments further improved the MedViT model’s accuracy to 0.905 (95% CI 0.893–0.916), showcasing its robustness in handling domain shift.

**Conclusion:**

Advanced neural network architectures like MedViT and expert domain knowledge on the target dataset significantly enhance the performance of MRI sequence classification models under domain shift conditions. By combining the strengths of CNNs and transformers, hybrid architectures offer enhanced robustness for reliable automated MRI sequence classification in diverse research and clinical settings.

**Key Points:**

***Question*** *Domain shift between adult and pediatric MRI data limits deep learning model accuracy, requiring solutions for reliable sequence classification across diverse patient populations.*

***Findings*** *The MedViT model outperformed ResNet-18 in pediatric imaging; expert domain knowledge adjustment further improved accuracy, demonstrating robustness across diverse datasets.*

***Clinical relevance*** *This study enhances MRI sequence classification by leveraging advanced neural networks and expert domain knowledge to mitigate domain shift, boosting diagnostic precision and efficiency across diverse patient populations in multicenter environments.*

**Graphical Abstract:**

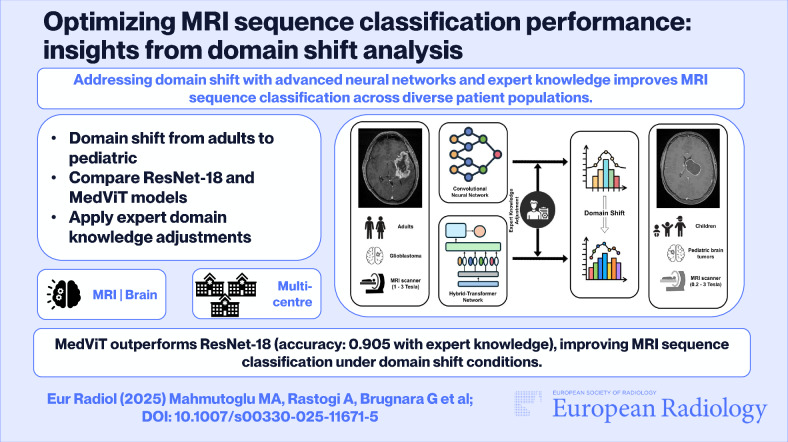

## Introduction

MRI sequence classification poses significant challenges in multicenter studies due to variability in imaging protocols. Sequence classification becomes particularly important in large cohort studies, especially in multicenter settings where imaging protocols can vary significantly. Unfortunately, relying on metadata stored in DICOM headers for labeling of medical images is often unreliable or impractical due to a lack of standardization across institutions, devices, and medical personnel [1]. These inconsistencies, coupled with the information loss from anonymization, often lead to missing sequence information, necessitating labor-intensive manual annotation.

Deep learning models, especially those based on convolutional neural networks (CNNs), have demonstrated potential in the automatic classification of MRI sequences [2–7]. However, these models often encounter difficulties when applied to data that deviates from the training set, a phenomenon known as domain shift [8–10]. This project addresses the problem of domain shift in classifying MRI sequences, specifically focusing on the shift from adult to pediatric data.

**Fig. 1:**
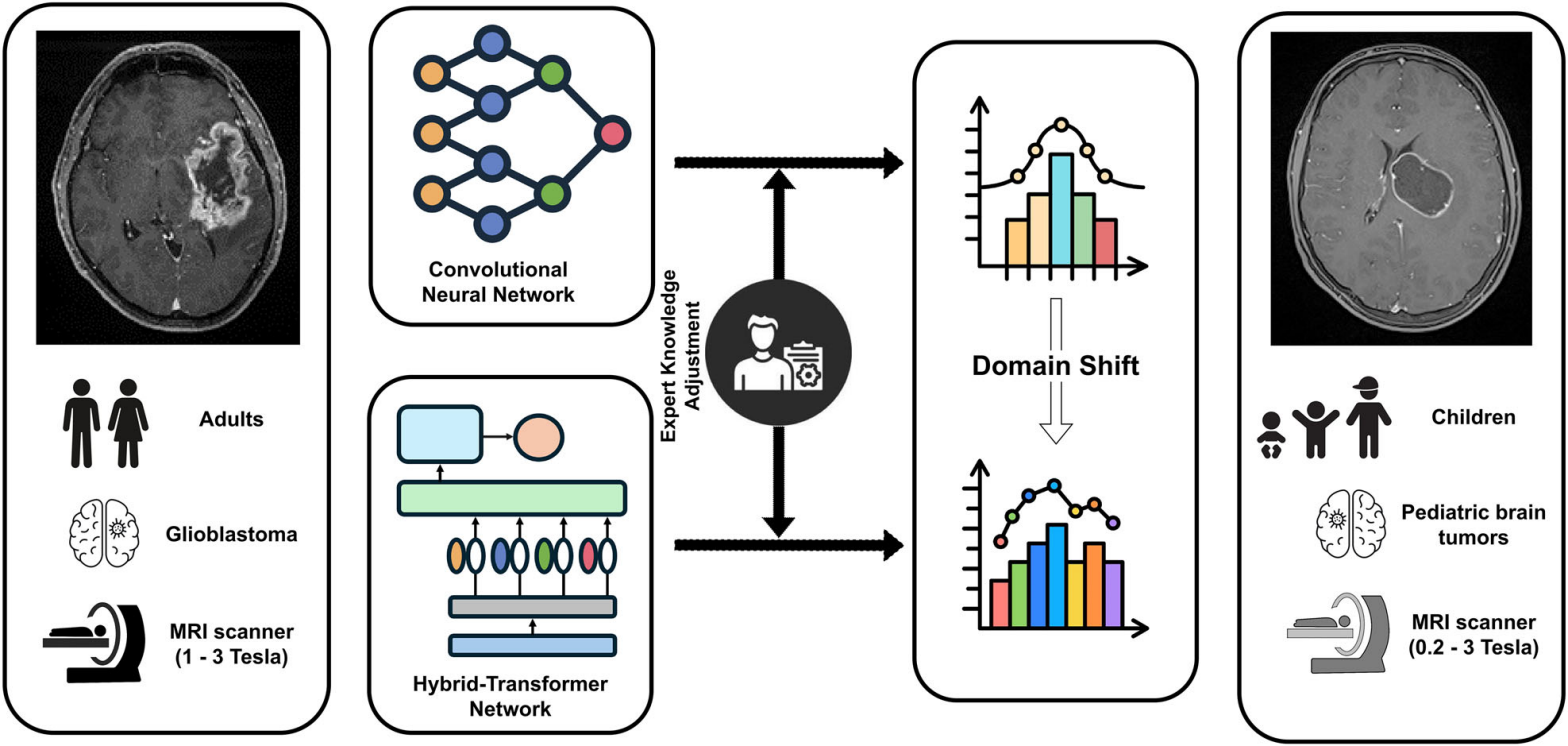
Experimental setup and analysis framework for evaluating domain shift in brain MRI classification. This figure illustrates the experimental setup to evaluate the impact of domain shift in brain MRI data and the adaptability of different neural network architectures. Convolutional Neural Networks (CNN) and Hybrid-Transformer Networks were trained on multicentric glioblastoma MRI data from adult patients (1–3-Tesla scanners) and tested on a multicentric pediatric brain tumor dataset (0.2–3-Tesla scanners). The comparison of the two architectures (CNN and Hybrid-Transformer Networks) aims to determine whether transformer-based networks adapt more effectively to domain shift in comparison to CNNs. The expert adjustment’s impact on both network types was also evaluated

The primary objective of this study is to evaluate the performance of a pretrained ResNet [11] model when subjected to domain shift induced by differences in field strength, sequences used, and patient demographics. We aim to identify new strategies that enhance the model’s performance in this new domain by exploring advanced Transformer-based neural network architectures and leveraging expert domain knowledge (Fig. 1).

We anticipate that the model’s performance will decline when applied to the pediatric dataset due to the domain shift. This expected drop serves as the basis for exploring performance enhancement strategies to mitigate the impact of domain shift.

To address this, we explored two key hypotheses:

1. **Utilization of modern models to mitigate domain shift**: Vision Transformers (ViTs) have shown significant promise in computer vision [12], frequently outperforming CNNs in handling distribution shifts. This performance can be further improved through specific modifications to the Transformer architecture [13]. Conversely, prior studies have emphasized that the self-attention mechanism, central to Transformer models, plays a key role in adapting to distribution shifts and that with proper tuning, CNNs can exhibit similar robustness [14, 15]. We investigated the MedViT [16], a modern CNN-Transformer hybrid model, based on the hypothesis that advanced hybrid model architectures may be more effective in managing domain shifts. The hybrid MedViT model was trained on the original adult data and evaluated on the pediatric dataset to determine whether advanced neural network architectures involving transformer layers are inherently better suited to adapt to domain variations.
2. **Incorporation of expert domain knowledge**: Pretrained models often struggle with domain shifts, particularly when there are changes in the sample distribution and target classes. This challenge is exacerbated when the test set includes classes unseen during training or some labels are missing, or their distribution changes compared to the training set. This project aimed to address the inherent difficulties posed by unbalanced sample distributions and qualitatively and quantitatively altered target classes in the new dataset. We hypothesized that expert domain knowledge could enhance model performance. In our case, the pediatric dataset contained fewer classes than the adult dataset used for the initial training., Therefore, we adjusted the model’s decision-making process to ignore labels absent from the pediatric test set. This adjustment aimed to align the model’s classification task more closely with the characteristics of the new data, which in turn expects prior knowledge of which labels are already included in the imaging protocols.

Overall, this research contributes to enhancing the robustness and reliability of pretrained neural networks in medical imaging as proof-of-concept. While the translation of deep learning techniques from research into healthcare has been slow in recent years [17], by addressing the effect of domain shift as a significant challenge, we aim to improve the accuracy and reliability of automated MRI sequence classification across diverse patient populations.

## Materials and methods

### Dataset

Retrospective multicenter brain MRI data, comprising 8544 examinations and 63,327 sequences from 2179 glioblastoma patients, were collected from 249 hospitals utilizing 29 different scanner types. Four glioblastoma cohorts included in this retrospective study, namely (1) institutional cohort (approved by the local ethics committee, reference S-784 2018), and the evaluation of the (2) CENTRIC [18], (3) CORE [19] and (4) EORTC-26101 [20, 21] cohorts was granted through an external research project (reference ERP-263 and ERP-362) with the European Organization for Research and Treatment of Cancer (EORTC). This collective dataset was employed recently to develop a network based on the ResNet-18 architecture for distinguishing nine different MRI sequence types [22], which includes the following MRI sequence types: T1-weighted, postcontrast T1-weighted (CT1), T2-w, fluid-attenuated inversion recovery (FLAIR), susceptibility-weighted imaging (SWI), apparent diffusion coefficient (ADC), diffusion-weighted imaging (DWI) sequences with low b-values (Low-B-DWI) and DWI sequences with high b-values (High-B-DWI). Furthermore, a “T2*/DSC-related” class was included, encompassing gradient recalled echo T2*-weighted imaging (T2*) and dynamic susceptibility contrast (DSC)-related sequence types.

To introduce a significant domain shift, an MRI dataset was used, comprising pediatric central nervous system tumors, which was collected from 51 centers in the context of the Molecular Neuropathology 2.0 (MNP 2.0) study [23, 24]. MRI data from 667 patients with a preoperative MRI exam were available, yielding a total of 2397 MRI sequences. Visual quality assessment of all available sequences was conducted, resulting in the exclusion of 14 sequences. Exclusion criteria were (1) foreign fixation devices causing metal artifacts (*n* = 3), (2) unclear label in non-anatomical sequence (*n* = 10) and (3) motion artifacts (*n* = 1). After exclusion of unsuitable sequence types, 2383 MRI sequences remained, consisting of T2* (*n* = 56), T1 (*n* = 574), T2 (*n* = 592), contrast-enhanced T1 (*n* = 490), FLAIR (*n* = 502) and SWI (*n* = 169).

### MRI sequence labeling

The preprocessing of the training dataset was outlined in Mahmutoglu et al [22]. For the test set, sequence labeling was done semi-automatically by G.B., where metadata was first used to depict sequence names from DICOM metadata and subsequently visually confirmed. This was followed by a second visual inspection on midslices of each volume by M.A.M.

### Deep learning model development

#### Benchmark model

The ResNet-18 model from Vieira de Mello et al [2] was chosen as a Benchmark model for several reasons: (1) direct comparability with the ResNet-18 model proposed in this study due to training on single MRI slices, (2) trained on adult brain MRIs, (3) encouraging results in their initial experiments on publicly available datasets, (4) targets the identification of anatomical sequence types which are mostly available in pediatric MRI protocols, (5) designates scans as “Other” class if the MRI sequence cannot be classified as one of the anatomical sequences.

#### ResNet-18 model

The training data (10,771 exams, 43,601 MRIs) was split into balanced groups across institutions using “scikit-learn” library’s stratified split method (train fold ~64%, validation fold ~16%), analogous to Mahmutoglu et al [22]. The network training was performed with Pytorch (version 1.12.1). Training data augmentation was performed with the Monai package (https://monai.io, version 0.8.0) by resizing the images to 200 × 200 × 1 pixels, applying Gaussian noise (mean = 0, std = 0.1), and normalizing the intensity. Class weights were assigned according to the class imbalance, and the optimization was done using the Adam algorithm [25] with a batch size of 32 and cross-entropy as the loss function. Learning rate was set to 0.01. The ResNet-18 model is trained for 200 epochs.

#### MedViT model

Analogous to ResNet-18 model training, same preprocessing and data augmentation steps are done for training the MedViT model. MedViT [16] is a novel hybrid CNN-transformer architecture for medical image classification, which achieved state-of-the-art accuracy and robustness on the standard large-scale collection of 2D biomedical datasets. Since MedViT expects a 3-channel RGB input, which was not provided by MRI images, the same midslice MRI image was copied to all 3 channels.

### Model training and testing

Training of ResNet-18 and MedViT models involved nine MRI sequence classes (T1, T2, CT1, FLAIR, ADC, SWI, Low-B-DWI, High-B-DWI, T2*/DSC-related). Test dataset included six classes (T1, T2, CT1, FLAIR, SWI, T2*) and the Benchmark model [2] was trained to differentiate four anatomical MRI classes (T1, T2, CT1, FLAIR) with defining the rest of the predictions as “Other.” To achieve accordance for model comparison and simplify the classification results across all statistical steps, we focused on four anatomical MRI sequence types (T1, T2, CT1, FLAIR) as the most relevant and frequently occurring types in pediatric MRI protocols. For this, the remaining sequence types (T2* and SWI) were grouped together as the “Other” class. In return, if ResNet-18 and MedViT models predicted different outputs than the anatomical sequence types, these were also labeled as the “Other” class.

### Expert domain knowledge adjustment

Performance analysis of pretrained models initially covered all available images in the test dataset. For comparison purposes, we employed “expert domain knowledge adjustment.” This adjustment assumes that MRI protocols have been predefined by an expert, meaning the types of sequences present in the data are already known. Although the models were pretrained on a wide range of MRI sequence types, the predictions can be fine-tuned to align with the new dataset during testing without updating the model weights. Given that the neural network model has been trained to recognize a wider range of MRI sequence types (nine in this case) than those present in the institutional dataset (six in this case), it is possible to exclude or censor the MRI sequences that were part of the model’s training but are not relevant to the institutional data during the prediction phase. This adjustment was applied to pretrained ResNet-18 and MedViT models to evaluate their performance in detecting out-of-distribution data and managing partial label shifts. We refer to this method as “expert domain knowledge adjustment,” which involves setting the softmax output of missing labels to “-∞”. For instance, if the models generate a softmax output for nine labels, and only six are relevant according to expert domain knowledge, the outputs for the irrelevant three labels are set to “-∞”, ensuring they are not predicted. Instead, the second-highest softmax value is chosen as the prediction if the choice with the highest value was set to “-∞”. This adjustment serves to incorporate “expert domain knowledge” at the prediction stage to optimize model performance.

The benchmark model did not require adjustment since it was trained on fewer MRI classes than the target dataset.

### Statistical analysis

The models were evaluated using several performance metrics: accuracy, macro average class accuracy (macro accuracy), weighted F1-score, macro average F1-score, weighted specificity, and macro average specificity. Class-specific evaluations were conducted using F1-score, specificity, precision, and recall. Bootstrapping with *n* = 1000 was employed to calculate 95% confidence intervals (CI). To compare the accuracy between models, one-way ANOVA was applied on bootstrapped results with post hoc analysis using Tukey’s HSD correction. A *p*-value < 0.05 was considered significant for all analyses. Confusion matrices were visualized for all models and for each MRI sequence class.

## Results

Data preprocessing and inclusion/exclusion criteria were described in Mahmutoglu et al [22]. Figure 1 illustrates the simplified experimental setup used to evaluate the effects of domain shift in brain MRI data and the adaptability of different neural network architectures. Predictions for each model were compared to ground truth and bootstrapped results (*n* = 1000) were compared using one-way ANOVA and post hoc Tukey’s HSD. Performance analysis of the models on the pediatric test dataset revealed better performance of the MedViT model compared to the ResNet-18 and Benchmark models (*p* < 0.001 each). Model comparison results are shown in Table 1.

**Table 1:** Results of an ANOVA test and Tukey’s HSD post hoc analysis comparing the performance of three models

ANOVA	sum_sq	df	F	PR (> F)	
Variable	13.779072	2.0	110143.564927	< 0.001	
Residual	0.187464	2997.0	-	-	

Tukey’s HSD	group1	group2	meandiff	*p*-adj	reject
	MedViT	Benchmark	−0.1644	< 0.001	True
	MedViT	ResNet-18	−0.1023	< 0.001	True
	Benchmark	ResNet-18	0.0621	< 0.001	True

MedViT, Benchmark, and ResNet-18. The ANOVA results show a significant effect of the model on performance (*p* < 0.001). The Tukey’s HSD analysis pairwise comparisons are statistically significant, indicating that the models differ significantly in their performance

**Fig. 2:**
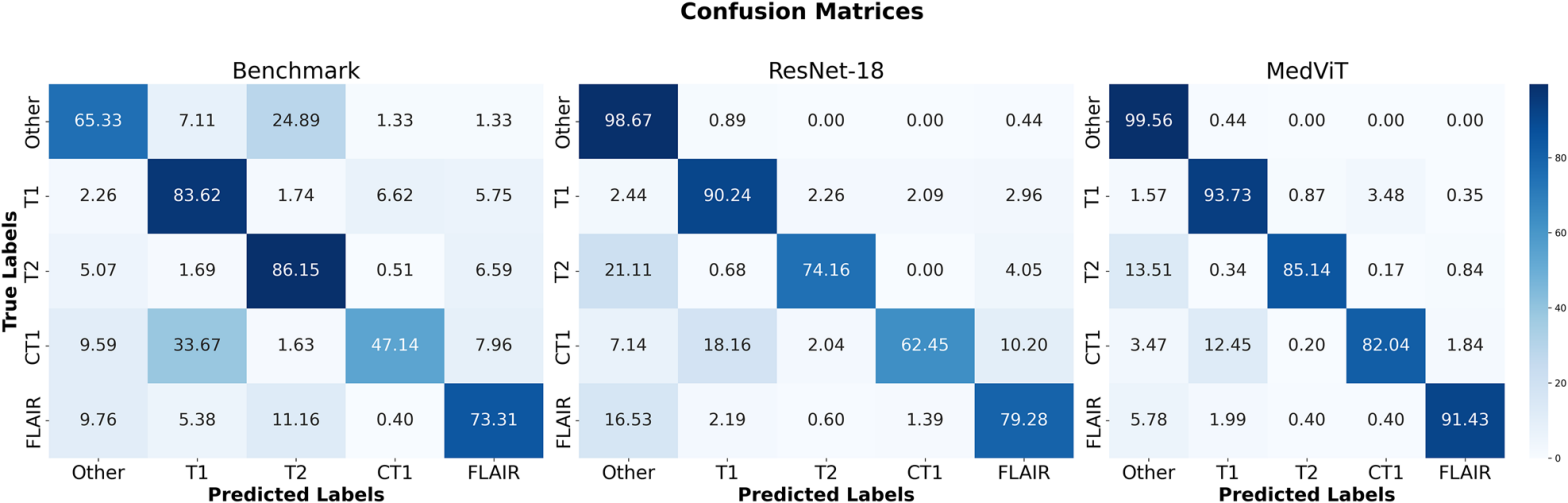
Classification results on the pediatric test set. Confusion matrices indicate the true and predicted values for each MRI sequence class, comparing the classification performance of three models (Benchmark, ResNet-18 and MedViT) across different MRI sequence types (T1, T2, CT1, FLAIR and Other). The matrices illustrate the percentage of correct and incorrect classifications made by each model

Confusion matrices for each model are illustrated in Fig. 2 to emphasize prediction differences for each MRI class.

**Fig. 3:**
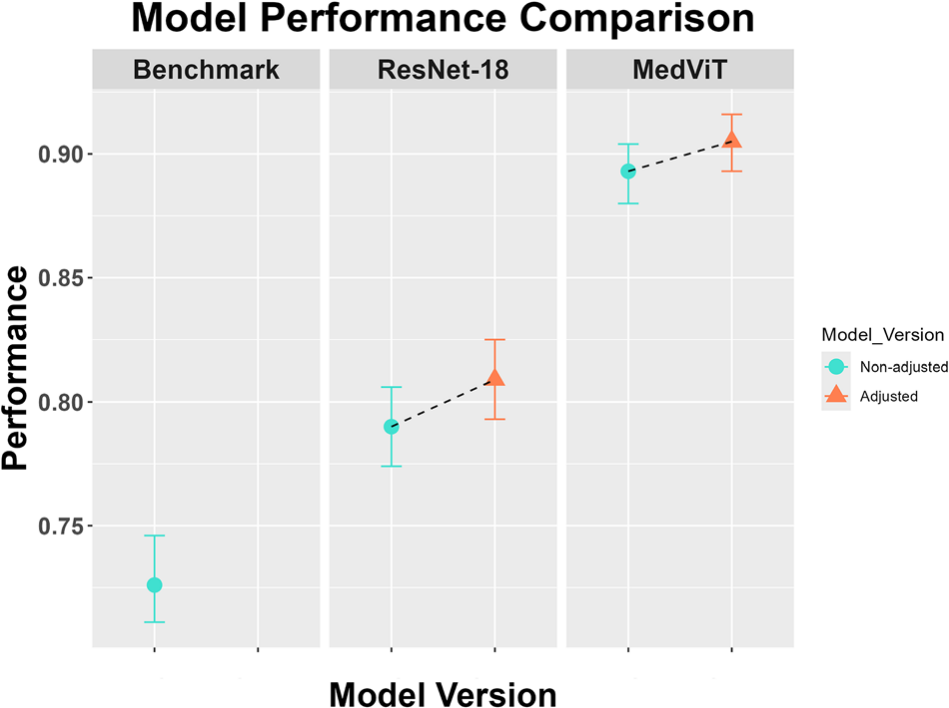
Comparison of model performance across three different models: Benchmark, ResNet-18 and MedViT. Performance metrics are plotted on the *y*-axis (accuracy with confidence intervals), with the models on the *x*-axis. The “Adjusted” (red circles) refers to models that have undergone expert domain knowledge adjustment, while “Non-adjusted” (blue triangles) represents the original model configurations. The comparison highlights the performance improvements gained through expert domain knowledge adjustments

Performance analysis of the models on the pediatric test dataset revealed better performance of the MedViT model compared to the ResNet-18 model and the Benchmark model in terms of accuracy (*p* < 0.001 each). The accuracy of the MedViT model was 0.893 (95% CI 0.880–0.904) on the test dataset, compared to the ResNet-18 model with 0.790 (95% CI 0.774–0.806) and benchmark model with 0.726 (95% CI 0.711–0.746) as shown in Table 2 and Fig. 3 (referred to as “Non-adjusted”). The “expert domain knowledge adjustment” revealed slightly better performance for MedViT and ResNet-18 models compared to the native models in pediatric test dataset (*p* < 0.001 each). The accuracy of the adjusted MedViT model was 0.905 (95% CI 0.893–0.916) on the pediatric dataset, compared to the ResNet-18 model with 0.809 (95% CI 0.793–0.825), as shown in Table 2 and Fig. 3 (referred to as “Adjusted”).

**Table 2:** F1-score and accuracy of ResNet-18 and MedViT models compared to the benchmark model across various MRI sequence types (T1, T2, CT1, FLAIR and Other)

	Benchmark	Non-adjusted		Adjusted		F1-Score
		ResNet-18	MedViT	ResNet-18	MedViT	
**Sequence type**						
Other	0.575	0.631	0.767	0.695	0.819	
T1	0.755	0.865	0.907	0.864	0.906	
T2	0.828	0.831	0.913	0.870	0.934	
CT1	0.602	0.751	0.879	0.751	0.880	
FLAIR	0.748	0.802	0.940	0.798	0.941	
**Accuracy**	0.728	0.790	0.893	0.809	0.905	
**Macro average**	0.702	0.776	0.881	0.795	0.896	
**Weighted average**	0.723	0.798	0.896	0.812	0.907	

The results are shown before (non-adjusted) and after expert domain knowledge adjustment (adjusted)

## Discussion

The results of this study demonstrate that domain shift presents a significant challenge in MRI sequence classification, particularly in transitioning from adult to pediatric data. Our investigation revealed that deep learning models, while promising, require careful adaptation to new domains in medical imaging, as evidenced by the drop in performance observed when pretrained models were applied to the pediatric dataset without adjustments. By addressing domain shift through Transformer-based model architectures and expert domain knowledge, we achieved improvements in classification performance.

This study utilized diverse patient populations, with heterogeneous adult cohorts for training and pediatric cohorts for testing, to improve generalizability. Additionally, image augmentation techniques were applied during training to minimize overfitting to specific scanner attributes or patient demographics.

### Domain shift and its impact

Domain shift occurs when the training data does not accurately represent the distribution of the test data, leading to decreased model accuracy on new, unseen data. This issue is particularly pronounced in medical imaging, where variations in protocols across institutions can significantly affect model performance. Standard deep learning models, typically designed under the assumption of identical training and test data distributions, struggle with such shifts.

As anticipated, both the ResNet-18 and MedViT models experienced performance degradation when applied to the pediatric dataset, which differed substantially from the adult data used during training. This outcome aligns with previous research highlighting the susceptibility of convolutional neural networks (CNNs) to domain shifts, particularly in medical imaging, where patient demographics, imaging protocols, and field strength can vary across institutions. The pediatric dataset presented fewer MRI sequence classes, as well as anatomical and physiological differences that influenced image acquisition characteristics, contributing to this domain shift. Notably, the performance decline was more pronounced in the ResNet-18 and benchmark models, which were less equipped to handle these variations.

Besides the domain shift, class imbalances might have affected the model performance. For example, about 55% of the training set belonged to the so-called “Other” class, whereas only ~9% of the pediatric test set consisted of “Other” class. While class imbalances might bias the model performance, it also reflects a realistic scenario, as models trained on external/heterogeneous datasets often encounter different class distributions when applied to internal data, as in our study. A model’s ability to adapt its decision-making to the imbalanced target dataset can be advantageous, given that fine-tuned models are prone to exhibit lower accuracies in real-world situations.

#### Advantages of transformer and hybrid models

Recent studies have shown that transformer models can better adapt to new data compared to traditional CNNs due to their self-attention mechanisms, which allow for more flexible feature representation. For instance, DA-DETR [26], ConvFormerSR [27] and CTFSL [28] hybrid models have demonstrated improved performance in various cross-domain tasks by combining the strengths of both CNNs and transformers.

The superior performance of the MedViT model over the ResNet-18 and benchmark models, both before and after expert domain knowledge adjustment, supports our hypothesis that modern hybrid architectures are better suited for handling domain shifts. Specifically, MedViT’s incorporation of Transformer layers—known for their global attention mechanisms—appears to enhance the model’s robustness to distributional changes [29]. The ability of Transformers to capture long-range dependencies likely contributed to their improved adaptation to the pediatric data, which exhibited variations in sequence characteristics not seen in the training set.

Interestingly, the MedViT model consistently outperformed the ResNet-18 model, even without domain knowledge adjustment. This suggests that hybrid CNN-Transformer models offer an inherent advantage over traditional CNNs in classifying MRI sequences under domain shift conditions. The findings align with previous research suggesting that Transformers, and by extension hybrid architectures, may be better equipped to handle out-of-distribution data [13, 15].

### Incorporation of expert domain knowledge

Incorporating expert domain knowledge significantly improved model performance for both MedViT and ResNet-18. By constraining the models’ predictions to the relevant sequence classes in the pediatric dataset, we effectively reduced the search space and aligned model predictions with clinical expectations. This adjustment led to a marked increase in accuracy for both models, with MedViT achieving an accuracy of 0.905 compared to 0.809 for ResNet-18.

Our results indicate that the T2 and “Other” classes benefited the most from “expert domain knowledge adjustment,” regardless of the model (see Table 2). In the ResNet-18 model, accuracy for the “Other” class improved by 6.4% and by 3.9% for T2, while other classes remained stable or showed slight declines. Similarly, the MedViT model exhibited a 5.2% increase in accuracy for the “Other” class, a 2.1% gain for T2, and minimal changes (0.1%) for CT1 and FLAIR. This improvement can be attributed to the model’s refined focus on available MRI sequences during the prediction step, facilitated by the introduced “expert domain knowledge adjustment.” Additionally, since many MRI sequences categorized under the “Other” class are derived from T2-weighted imaging (e.g., DWI, SWI, T2*, perfusion parameters), this targeted adjustment likely enhanced the model’s performance, particularly for T2-weighted images. This finding underscores the importance of domain-specific knowledge when applying pretrained models to new datasets. In clinical settings, it is often feasible to define MRI protocols or sequence types ahead of time, allowing for the strategic use of expert knowledge to refine model outputs. This approach could be particularly useful when working with diverse or incomplete datasets, where label shifts and unseen classes pose challenges.

In contexts where MRI protocols are predefined, awareness of the present sequence types can significantly improve classification accuracy, as evidenced in our study. However, the availability of such expert domain knowledge adjustment of pretrained models is not always guaranteed, especially in high-throughput data environments where detailed protocol information might be lacking. Consequently, the MedViT model’s strong performance highlights the inherent robustness of hybrid models, even in the absence of human insights.

### Limitations

This study has several limitations. The comparison was limited to the ResNet-18 model, which is well-established but may not represent the full spectrum of available models. Future research should explore a broader range of models and datasets to validate the findings further. MedViT was originally compared to ResNet architectures [16], and the benchmark model [2] used in this study also relied on ResNet backbone. This alignment led to our focus on ResNet-18 as the primary network architecture for comparison in this analysis. Additionally, the pediatric dataset used for testing may not fully represent all possible variations, limiting the generalizability of the findings. Expanding the scope of testing to include more diverse datasets and imaging conditions would provide a more comprehensive evaluation of the model’s performance and adaptability. Finally, unbalanced distributions of the MRI sequence types in the training and test sets might have affected the results while simulating a realistic scenario in daily clinical and research settings.

## Conclusion

Our study highlights the significant impact of domain shift on MRI sequence classification and demonstrates the potential advantages of modern hybrid models, such as MedViT, in addressing this challenge. By combining the strengths of CNNs and transformers, hybrid architectures offer enhanced robustness in handling domain shifts. Furthermore, incorporating expert domain knowledge can further refine model performance, although its feasibility may vary depending on the availability of detailed protocol information. Future research should continue to explore and validate advanced model architectures and strategies to improve the accuracy and reliability of automated medical imaging systems across diverse clinical scenarios.

## Supplementary information

ELECTRONIC SUPPLEMENTARY MATERIAL

## Abbreviations

CI: Confidence intervals
CNNs: Convolutional neural networks
CT1: Postcontrast T1-weighted
DSC: Dynamic susceptibility contrast
DWI: Diffusion-weighted imaging
FLAIR: Fluid-attenuated inversion recovery
SWI: Susceptibility-weighted imaging
