# Supplementary material for: Optimizing MRI sequence classification performance: insights from domain shift analysis
Source: Eur Radiol. 2025 May 26;35(11):6710–8. doi: 10.1007/s00330-025-11671-5 (PMC12559157; doi:10.1007/s00330-025-11671-5)
Supplement: Supplementary file 1 — ELECTRONIC SUPPLEMENTARY MATERIAL [file 330_2025_11671_MOESM1_ESM.pdf]

Optimizing MRI Sequence Classification Performance: Insights from Domain Shift Analysis

ELECTRONIC SUPPLEMENTARY MATERIAL

Supplementary Figure 1: MRI manufacturer and field strength distribution in the test set (MNP cohort). For the dataset characteristics of the training set including manufacturer and field strength distribution, please refer to the Supplementary Appendix of (Mahmutoglu et al. 2024).

**(Mahmutoglu et al. 2024)MRI scanners**	**Count**	**Percentage**
**Siemens**	**395**	**49.9%**
Unknown	6	1.0%
Aera	93	15.2%
Avanto	155	25.3%
Biograph	1	0.2%
Espree	10	1.6%
Harmony	1	0.2%
Harmony Expert	3	0.5%
Magnetom Altea	1	0.2%
Magnetom Essenza	3	0.5%
Prisma	9	1.5%
Skyra	51	8.3%
Sonata	8	1.3%
SonataVision	2	0.3%
TrioTim	36	5.9%

Verio	16	2.6%
**Philips**	**147**	**18.6%**
Unknown	6	1.0%
Achieva	57	9.3%
Achieva dStream	6	1.0%
Ingenia	48	7.8%
Intera	21	3.4%
Panorama HFO	9	1.5%
**GE**	**24**	**3.0%**
Discovery MR750	6	1.0%
Discovery MR750w	7	1.1%
Optima MR360	2	0.3%
Signa Excite	2	0.3%
Signa Explorer	1	0.2%
Signa HDxt	6	1.0%
**Toshiba**	**7**	**0.9%**
Titan	4	0.7%
Vantage Elan	3	0.5%
**Hitachi**	**1**	**0.1%**
AIRIS Mate	1	0.2%
**Unknown**	**1**	**0.1%**

**MRI field strength**		
0.2 Tesla	1	0.2%
0.6 Tesla	1	0.2%
1 Tesla	14	2.3%
1.5 Tesla	414	67.5%
3 Tesla	182	29.7%
Unknown	1	0.2%

Supplementary Table 2: Patient demographics of the test set (MNP cohort). For patient demographics of the training set including manufacturer and field strength distribution, please refer to the Supplementary Appendix of (Mahmutoglu et al. 2024).

**Patient demographics**	
**Sex**	**Percentage**
Male	55.1%
Female	44.9%
**Age**	**Years (mean ±std)**
Male	8.2 ± 5.5
Female	7.7 ± 5.2

Preprocessing and Model Training

Training set

Training and preprocessing steps were analogous to **(Mahmutoglu et al. 2024)**.

The training set incorporated four glioblastoma cohorts: an institutional cohort from Heidelberg, Germany (approved by the local ethics committee under reference S-784 2018), the multicenter phase 2 CORE trial (CORE cohort; NCT00813943; (Nabors et al. 2015)), the multicenter phase 3 CENTRIC trial (CENTRIC cohort; NCT00689221; (Stupp et al. 2014)), and the multicenter phase 2 and phase 3 EORTC-26101 trial (NCT01290939; (Wick et al. 2017; Wick et al. 2016)). Access to the CENTRIC, CORE, and EORTC-26101 data was granted via external research projects (ERP-263 and ERP-362) with the EORTC.

Data Preprocessing for Training Set

All DICOM files were converted to the NIfTI format (http://nifti.nimh.nih.gov) using dcm2niix (https://github.com/rordenlab/dcm2niix), MRIConvert (https://lcni.uoregon.edu/downloads/mriconvert/mriconvert-and-mcverter), or MRIcron (https://www.nitrc.org/projects/mricron). Ground-truth labeling was performed by a neuroradiologist.

When 4D DWI data were available (56% of all DWI data), low and high b-value volumes were separated post-conversion. Each volume was reoriented to the MNI152 standard space, with neck and lower head parts removed. Using FSL tools (https://fsl.fmrib.ox.ac.uk/fsl/fslwiki), the MRI was cropped to a 10 cm axial volume starting at the head vertex. The center-of-mass was calculated, and the midslice (at the center-of-mass) was selected for further analysis. This process produced 497 errors (0.8% of the data); the remaining 2D midslices (n = 63,327, one midslice per sequence) were retained, with no skull stripping or brain extraction at any stage.

A second quality check excluded 9618 slices (15%) for corrupt conversion (n=8497), unidentifiable signal (n=509), fractional DWI sequences exclusive to the CORE cohort (n=360), or midslice signals that did not match the initial 4D/3D labeling (n=252).

Model Training

Using the stratified 5-fold split from “scikit-learn” (Fabian Pedregosa et al. 2011), the entire dataset was partitioned into training/validation and test sets balanced by institution, patient, and MRI sequence type. 80% of the data was preserved for training and validation and was further split (again stratified 5-fold) into ~64% training and ~16% validation.

In total, 4634 T1, 3206 cT1, 7557 T2, 7941 FLAIR, 6684 DWI (3047 Low-B-DWI and 3637 High-B-DWI), 4656 ADC, and 878 SWI sequences were included for training and validation. An additional “T2*/DSC-related” category (n=16830) consisted of 1278 T2* images plus various perfusion parameter volumes: 3197 CBV, 1637 CBF, 1534 MTT, 1534 TTP, 1534 TMAX, 1533 K2, and 4583 unspecified DSC perfusion sequences (PERF).

Training parameters

Model training parameters and transforms were constant across ResNet-18 and MedViT models, as outlined in the main manuscript:

Transforms:

- Resize: 200x200x1 pixels
- Gaussian noise (mean=0, std=0.1)
- Intensity normalization

Optimizer: Adam **(Kingma and Ba 2014)**

Loss function: Cross entropy loss

Batch size: 32

Learning rate: 0.01

Publication bibliography

Fabian Pedregosa; Gaël Varoquaux; Alexandre Gramfort; Vincent Michel; Bertrand Thirion; Olivier Grisel et al. (2011): Scikit-learn: Machine Learning in Python. In *Journal of Machine Learning Research* 12 (85), pp. 2825–2830. Available online at http://jmlr.org/papers/v12/pedregosa11a.html.

Kingma, Diederik P.; Ba, Jimmy (2014): Adam: A Method for Stochastic Optimization. In *CoRR*. DOI: 10.48550/arXiv.1412.6980.

Mahmutoglu, Mustafa Ahmed; Preetha, Chandrakanth Jayachandran; Meredig, Hagen; Tonn, Joerg-Christian; Weller, Michael; Wick, Wolfgang et al. (2024): Deep Learning-based Identification of Brain MRI Sequences Using a Model Trained on Large Multicentric Study Cohorts. In *Radiology. Artificial intelligence* 6 (1), e230095. DOI: 10.1148/ryai.230095.

Nabors, L. Burt; Fink, Karen L.; Mikkelsen, Tom; Grujicic, Danica; Tarnawski, Rafal; Nam, Do Hyun et al. (2015): Two cilengitide regimens in combination with standard treatment for patients with newly diagnosed glioblastoma and unmethylated MGMT gene promoter: results of the open-label, controlled, randomized phase II CORE study. In *Neuro-oncology* 17 (5), pp. 708–717. DOI: 10.1093/neuonc/nou356.

Stupp, Roger; Hegi, Monika E.; Gorlia, Thierry; Erridge, Sara C.; Perry, James; Hong, Yong-Kil et al. (2014): Cilengitide combined with standard treatment for patients with newly diagnosed glioblastoma with methylated MGMT promoter (CENTRIC EORTC 26071-22072 study): a multicentre, randomised, open-label, phase 3 trial. In *The Lancet. Oncology* 15 (10), pp. 1100–1108. DOI: 10.1016/S1470-2045(14)70379-1.

Wick, Wolfgang; Gorlia, Thierry; Bendszus, Martin; Taphoorn, Martin; Sahm, Felix; Harting, Inga et al. (2017): Lomustine and Bevacizumab in Progressive Glioblastoma. In *The New England journal of medicine* 377 (20), pp. 1954–1963. DOI: 10.1056/NEJMoa1707358.

Wick, Wolfgang; Stupp, Roger; Gorlia, Thierry; Bendszus, Martin; Sahm, Felix; Bromberg, Jacoline E. et al. (2016): Phase II part of EORTC study 26101: The sequence of bevacizumab and lomustine in patients with first recurrence of a glioblastoma. In *JCO* 34 (15_suppl), p. 2019. DOI: 10.1200/JCO.2016.34.15_suppl.2019.
